# Supplementary material for: The C2H2 Zinc Finger Protein MaNCP1 Contributes to Conidiation through Governing the Nitrate Assimilation Pathway in the Entomopathogenic Fungus Metarhizium acridum
Source: J Fungi (Basel). 2022 Sep 7;8(9):942. doi: 10.3390/jof8090942 (PMC9505000; doi:10.3390/jof8090942)
Supplement: Supplementary file 1 [file jof-08-00942-s001.zip › jof-1910419-supplementary.pdf]

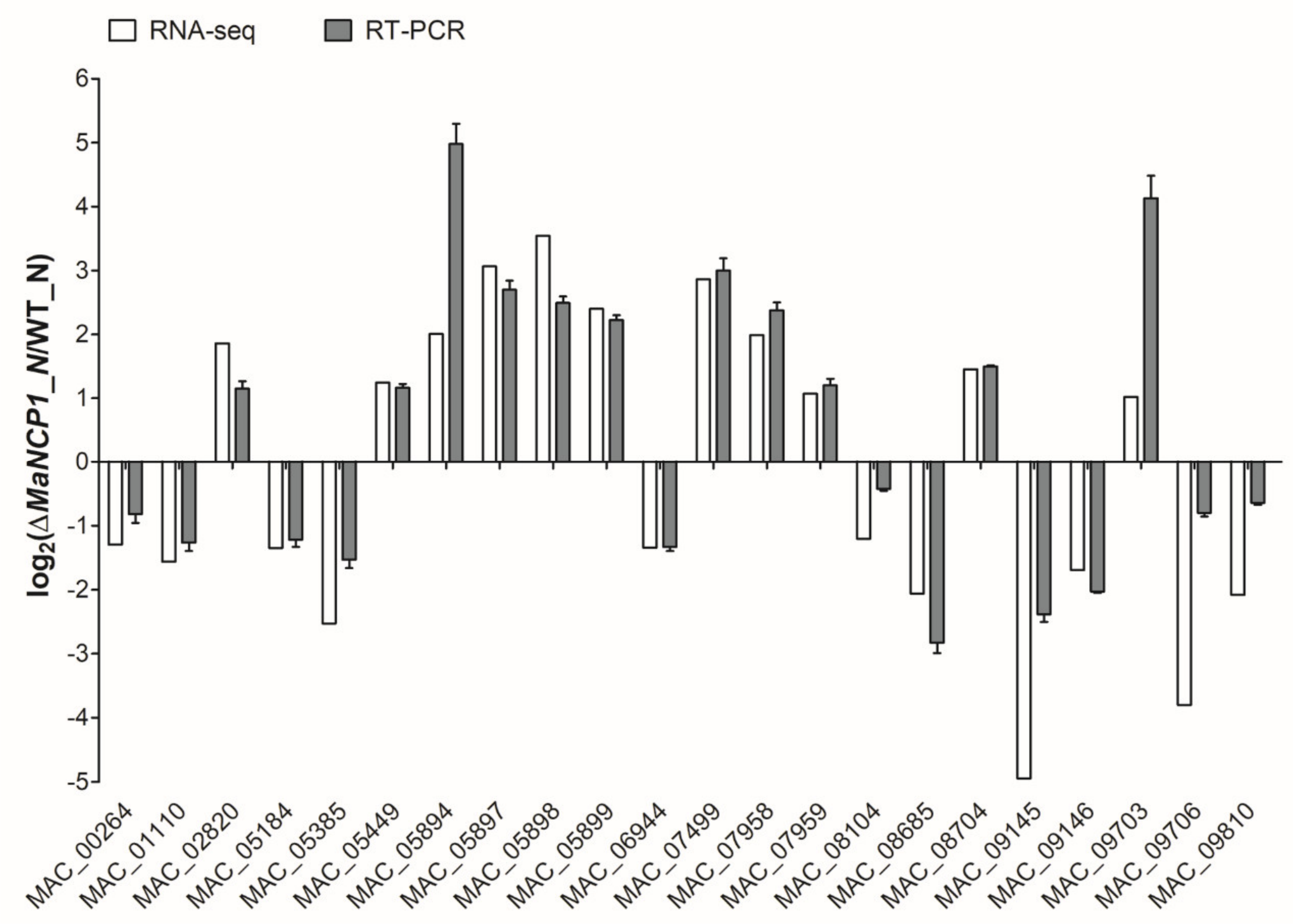

Figure S1. The verification of DEGs by qRT-PCR.

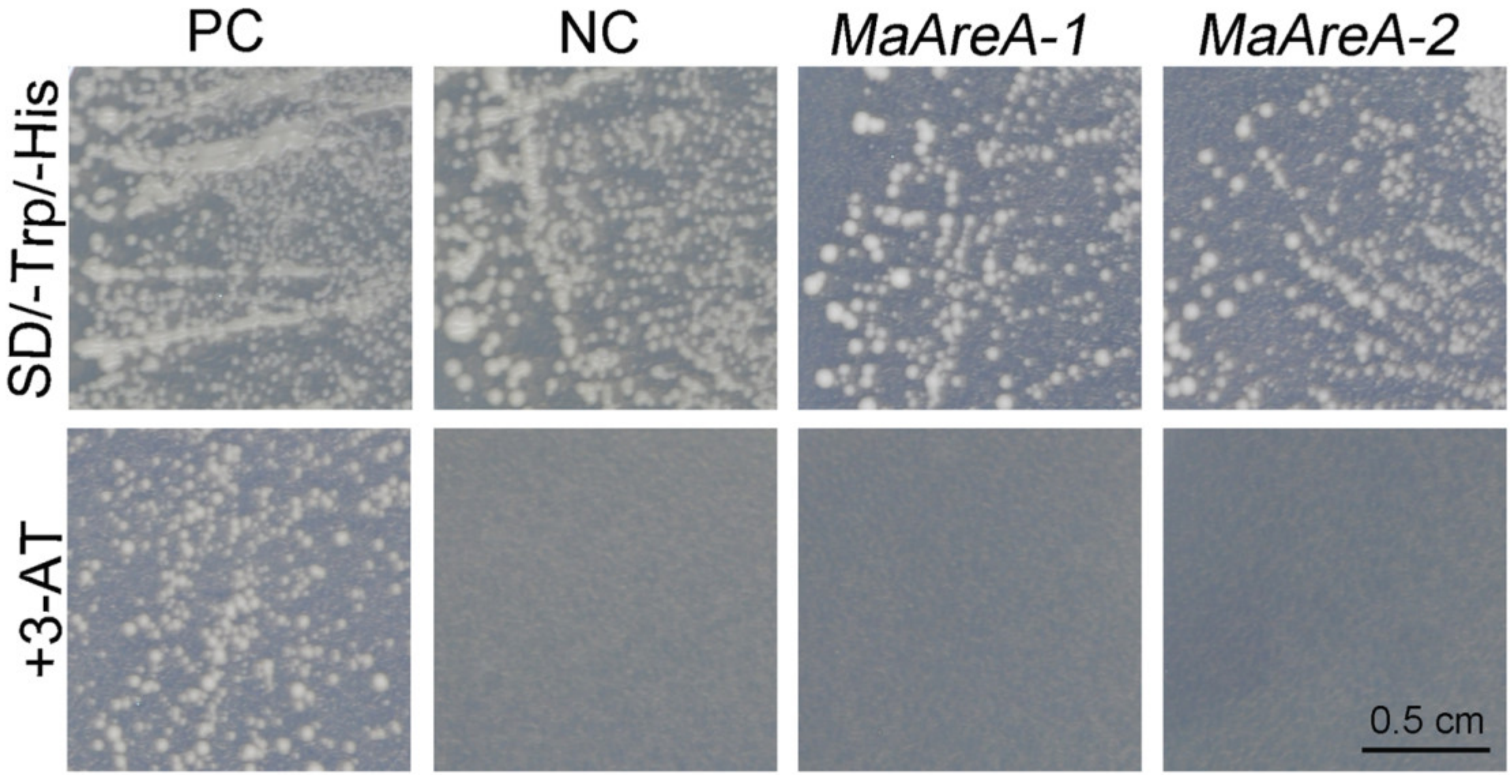

Figure S2. Screening of 3-AT background concentration of *MaAreA* promoter sequences. Y187(pHIS2-*MaAreA*-1) and Y187(pHIS2-*MaAreA*-2) were evenly spread on SD/-Trp/-His plate and SD/-Trp/-His plate with 8mM 3-AT to observe the growth of yeast. pGADT7-53 and pHIS2-53 as positive controls (PC), pGADT7-MaNCP1 and pHIS2 as negative control (NC).

Table S1. Primers used in this study

Primer	Sequence (5'-3')	Description	PCR efficiencies (%)
MaNCP1-F	ATGGACCCGTGGACTCAGGAG	For the cDNA sequences amplification	/
MaNCP1-R	TCATCCCGCATGCATGCCCAG		
MAC_03189-qF	CGTAGTTGATCCGTATGG	For qRT-PCR assay of genes related to nitrate assimilation	95.2
MAC_03189-qR	TAGAAGTAGAGTAGCACAGA		
MAC_08624-qF	AATGGTTGAAGAGGATAA		96.5
MAC_08624-qR	GTAACAGATGACACTATTG		
MAC_03493-qF	GTATCAACACCAAAGTCA		95.3
MAC_03493-qR	AATCGTCAATGGTTCTATA		
MAC_00032-qF	AAAGTTTGACGAGAGATTG		98.5
MAC_00032-qR	GTAGTATGAGTAGACCTGAA		
MAC_01108-qF	GCACAGTAACTTCTCAAC		100.3
MAC_01108-qR	TCATTGTCCTCTCCATAC		
MAC_06858-qF	TTTATGAGAAAGGATAGCA		99.2
MAC_06858-qR	TTAGACGGTTTCAATGTA		
MAC_04461-qF	CACCTCCATCTTTCTACT		96.4
MAC_04461-qR	GAACATAGCCGTCATAGT		
MAC_08384-qF	TACTGTCAACCTGTCCAT		99.8
MAC_08384-qR	ATTTCGCCATCAGACTTG		
MAC_01648-qF	GTATCCGTATTGTCAAGT		97.1
MAC_01648-qR	GGTCAAGAAGAATAACAC		
gpdh-qF	GACTGCCCGCATTGAGAAG		108.2
gpdh-qR	AGATGGAGGAGTGGGTGTTG		
MAC_00264-qF	CGAAACATTGATAAAGTC	For qRT-PCR assay of genes in transcriptome data	95.5
MAC_00264-qR	GCAAGTAATAGACATGAT		
MAC_01110-qF	TTGAAGTTTACAGAATGG		96.5
MAC_01110-qR	CTTGGGAAATAATATCGTA		

Table S1. (Continued)

MAC_02820-qF	ATCGTCCAGTTCTTTGAG		94.9
MAC_02820-qR	ATGCCGATTCTTGTAGTC		
MAC_05184-qF	CACCAGGAAGGATTATGA		96.8
MAC_05184-qR	AAGTCTCACCATGTTGTC		
MAC_05385-qF	TGGACTCATCACAAGAAG		95.4
MAC_05385-qR	GCTGTGGTTGTATAAGAC		
MAC_05449-qF	AGAATGCCAAGAATACCT		99.2
MAC_05449-qR	GAGAAGAGAGCCAGAAAG		
MAC_05894-qF	AAGAATATAGAATTGAGAGG		97.1
MAC_05894-qR	CTTCAAGTCTTCAAACAA		
MAC_05897-qF	TGACAATGGCATAACTCT		100.1
MAC_05897-qR	ATGAATGGTGTGTTGATG		
MAC_05898-qF	TTTACCAACAACAAGAAC		100.5
MAC_05898-qR	CTATCTACATTCCGAGTAT	For qRT-PCR assay of genes in transcriptome data	
MAC_05899-qF	ATGATAGTCTGCCTGATG		103.2
MAC_05899-qR	TTCTCCAATCCAAGTCTC		
MAC_06944-qF	CCTCTGCTATGCTGTTTA		96.4
MAC_06944-qR	CCTGGCTATTGTAACTCA		
MAC_07499-qF	CGGTGTATCTTTGAATCTC		98.1
MAC_07499-qR	ATTTCCTTGCCTTGTTTC		
MAC_07958-qF	ATCCAGCACATCCAGTAT		98.5
MAC_07958-qR	TAGGAGTTTGGGTGGTAG		
MAC_07959-qF	AAGACGATGCTATTGTAT		96.6
MAC_07959-qR	GTCAAATCTCTCTATATGC		
MAC_08104-qF	CTGATTCCATACTTTACG		94.5
MAC_08104-qR	TTATTCAAAGCCATACGA		
MAC_08685-qF	CGAATACCCAAGGATAAG		99.7
MAC_08685-qR	ATGCGATTGTCTCACTAT		

Table S1. (Continued)

MAC_08704-qF	CATTTCCAAGGTCATTAC	For qRT-PCR assay of genes in transcriptome data	100.4
MAC_08704-qR	CTATTGTCTCAGTCTTCT		
MAC_09145-qF	GATTCCTTCCATACTTCTCT		102.3
MAC_09145-qR	TCGTCCTCGTTATTGATG		
MAC_09146-qF	ATATTCAGCCAGGACAAG		101.2
MAC_09146-qR	TCGTTAGGAAGGGAGATT		
MAC_09703-qF	GTCATTACCTCTATTACG		99.8
MAC_09703-qR	TGAGATACCAAATAAGTG		
MAC_09706-qF	GCTCAAGTTCTCGTCAAG		97.5
MAC_09706-qR	TGGAAATCTCACACATCTG		
MAC_09810-qF	GCTGTTATTTCTATTCTTG		97.1
MAC_09810-qR	ATTTCTTCTGGTATATCG		
MaNCP1-AD-F	TGGCCATGGAGGCCAGTGATGGACCCGTGGACTCAGGAGC	For yeast expression vector construction. pHIS2-F/R, the universal primers of pHIS2 vector. T7/3' BD and T7/3' AD are the the universal primers of pGBKT7 and pGADT7 vectors, respectively.	/
MaNCP1-AD-R	GCTCGAGCTCGATGGATCTCATCCCGCATGCATGCCCAGA		
pHIS2-AreA1-F	GACTCACTATAGGGCGAATTCAACGAGTCCAAGCAAGTAATGA		
pHIS2-AreA1-R	ATAATGCCAGGAATTACTAGTGGACATTTTCGTCCATGCAGT		
pHIS2-AreA2-F	GACTCACTATAGGGCGAATTCACTGCATGGACGAAAATGTCC		
pHIS2-AreA2-R	ATAATGCCAGGAATTACTAGTGTTCTGTCATGGTGGGATCCAT		
pHIS2-F	TGCGGGCCTCTTCGCTATTAC		
pHIS2-R	AGGGCTTTCTGCTCTGTCATC		
T7	TAATACGACTCACTATAGGGCG		
3' BD	TTTTCGTTTTAAAACCTAAGAGTC		
3' AD	AGATGGTGCACGATGCACAG		
AreA-P-F	ATGGGCAACACTGACAACA	For AreA probe amplification	/
AreA-P-R	GCTTGCAACACAGAAGCAT		

Table S2. Differentially expressed genes (DEGs) in RNA-seq.

Gene ID	log2(Δ*MaNCM1_N*/WT_N)	Regulated	Q-value	P-value	Description
MAC_07957	4.006674414	Up	0.00016526	8.58E-05	Lactate dehydrogenase
MAC_05898	3.542727315	Up	3.77E-12	7.75E-13	Aminoadipate-semialdehyde dehydrogenase, putative
MAC_05897	3.065568103	Up	1.01E-13	1.88E-14	Hypothetical protein
MAC_07499	2.861857861	Up	7.70E-103	1.90E-104	Hypothetical protein
MAC_06275	2.650530604	Up	8.19E-05	3.99E-05	Acyltransferase, putative
MAC_05899	2.398991837	Up	0.000638775	0.000371322	ATP-binding cassette sub-family B member 5
MAC_09405	2.271015018	Up	4.75E-109	1.11E-110	Sorbitol dehydrogenase
MAC_07881	2.156421534	Up	3.18E-09	8.46E-10	Hypothetical protein
MAC_05894	2.006674414	Up	3.72E-06	1.45E-06	Oxidoreductase
MAC_07958	1.986496532	Up	2.02E-08	5.83E-09	Tyrosinase, putative
MAC_04934	1.960870725	Up	0.000254059	0.000136773	Hypothetical protein
MAC_02819	1.913732853	Up	2.76E-212	2.52E-214	2,3-dihydroxybenzoic acid decarboxylase dhbd
MAC_09447	1.861726078	Up	1.09E-12	2.17E-13	CRAL/TRIO domain protein
MAC_02820	1.860823548	Up	1.38E-05	5.77E-06	Fungal specific transcription factor, putative
MAC_05896	1.79572852	Up	3.78E-22	4.35E-23	RTA1 domain protein, putative
MAC_09188	1.714493663	Up	2.71E-05	1.21E-05	Hypothetical protein
MAC_06364	1.487776937	Up	0	0	Heat shock protein 30
MAC_00175	1.474845169	Up	4.40E-150	6.47E-152	Glucose dehydrogenase, putative
MAC_08704	1.45520506	Up	5.60E-58	2.43E-59	NADP-dependent leukotriene B4 12-hydroxydehydrogenase
MAC_06622	1.428907415	Up	1.74E-05	7.45E-06	Aspartic protease
MAC_04199	1.423809206	Up	2.42E-193	2.42E-195	Putative long-chain-fatty-aci--coa ligase FAA2
MAC_04042	1.405343517	Up	1.78E-139	3.01E-141	Oxidoreductase, 2-nitropropane dioxygenase family, putative
MAC_04217	1.372152788	Up	0	0	Heat shock protein 30
MAC_04018	1.354597718	Up	3.73E-06	1.45E-06	ABC multidrug transporter, putative
MAC_01184	1.352512998	Up	4.08E-57	1.78E-58	Ribose 5-phosphate isomerase
MAC_08674	1.331840883	Up	5.19E-14	9.34E-15	Hypothetical protein
MAC_03561	1.290038928	Up	0	0	Nonspecific lipid-transfer protein precursor
MAC_07885	1.286782334	Up	0.000538496	0.000308595	Hypothetical protein

Table S2. (Continued)

MAC_05815	1.267634492	Up	1.39E-08	3.93E-09	High-affinity nicotinic acid transporter
MAC_00321	1.266061043	Up	1.40E-09	3.59E-10	Hypothetical protein
MAC_05449	1.244779511	Up	6.98E-72	2.42E-73	Epoxide hydrolase 1
MAC_06731	1.237231197	Up	7.03E-90	1.99E-91	Thij/pfpi family protein
MAC_07930	1.235205973	Up	3.27E-09	8.72E-10	Sterol esterase precursor
MAC_01545	1.179633476	Up	4.22E-104	1.02E-105	Hypothetical protein
MAC_09815	1.175393653	Up	2.51E-15	4.11E-16	Fatty acid-binding protein FABP, putative
MAC_00172	1.151064324	Up	7.31E-09	2.01E-09	Hypothetical protein
MAC_02353	1.105164278	Up	4.42E-18	6.21E-19	Sodium/phosphate symporter, putative
MAC_03724	1.092593441	Up	9.73E-260	7.15E-262	Epoxide hydrolase
MAC_09768	1.086378419	Up	1.56E-60	6.30E-62	Amino-acid permease inda1
MAC_07959	1.072525094	Up	0	0	Hydantoinase/oxoprolinase, putative
MAC_06610	1.047701683	Up	2.12E-05	9.21E-06	Beta-1,3-endoglucanase
MAC_02607	1.041105458	Up	8.81E-113	2.00E-114	Hypothetical protein
MAC_09703	1.022271269	Up	0.000102202	5.07E-05	FAD binding domain-containing protein
MAC_00248	-1.021231582	Down	5.75E-11	1.31E-11	Hypothetical protein
MAC_02934	-1.031017876	Down	1.79E-14	3.10E-15	Glucose-methanol-choline oxidoreductase
MAC_04216	-1.056213292	Down	2.42E-26	2.38E-27	YT521-B-like splicing factor, putative
MAC_01714	-1.075009383	Down	5.59E-19	7.41E-20	Hypothetical protein
MAC_09120	-1.094411711	Down	2.59E-125	4.96E-127	Putative neurofibromin
MAC_02571	-1.097808903	Down	0	0	Endoglucanase, putative
MAC_07239	-1.121081133	Down	0.0008662	0.000517789	Reductase
MAC_06937	-1.148895294	Down	2.15E-11	4.72E-12	Hypothetical protein
MAC_01211	-1.170863771	Down	1.22E-05	5.09E-06	Aquaglyceroporin
MAC_08104	-1.204829691	Down	5.95E-12	1.25E-12	3-octaprenyl-4-hydroxybenzoate carboxy-lyase, putative
MAC_07328	-1.208338477	Down	0.000207271	0.000109606	Hypothetical protein
MAC_04761	-1.215853592	Down	2.18E-179	2.38E-181	Hypothetical protein
MAC_04073	-1.270723723	Down	0.000397674	0.000222496	Hypothetical protein
MAC_00264	-1.295292939	Down	6.45E-19	8.60E-20	Hypothetical protein

Table S2. (Continued)

MAC_09175	-1.316018833	Down	1.48E-54	6.83E-56	Protein CCC1, putative
MAC_06606	-1.334362504	Down	6.23E-65	2.27E-66	GMC oxidoreductase, putative
MAC_06944	-1.345310914	Down	7.84E-07	2.78E-07	P450 monooxygenase
MAC_05184	-1.348420544	Down	1.32E-07	4.23E-08	C2H2 type zinc finger domain protein
MAC_08413	-1.387604525	Down	8.43E-07	3.00E-07	Alpha/beta hydrolase fold family protein
MAC_01110	-1.561893648	Down	6.53E-14	1.19E-14	Cytochrome P450, putative
MAC_00247	-1.624830672	Down	2.16E-30	1.81E-31	Hypothetical protein
MAC_09146	-1.694040654	Down	2.59E-109	6.00E-111	Late sexual development protein
MAC_08685	-2.062134744	Down	2.66E-40	1.74E-41	Flavin containing amine oxidase, putative
MAC_05384	-2.067526528	Down	6.04E-42	3.78E-43	Laccase
MAC_09810	-2.080788427	Down	1.65E-08	4.70E-09	Membrane transporter
MAC_05385	-2.524707046	Down	2.27E-77	7.51E-79	Conidial pigment polyketide synthase pksp/Alb1
MAC_09706	-3.800680508	Down	0.000546937	0.000314162	Isopropanol dehydrogenase, putative
MAC_09145	-4.947521896	Down	3.55E-08	1.05E-08	Carboxylesterase family protein
MAC_04326	-5.400289784	Down	0	0	C2H2 type zinc finger domain protein

Table S3. Differentially expressed genes involved in nutrition utilization, growth and development, stress tolerance and pathogenicity

Gene ID	log2(Δ*MaNCM1_N*/WT_N)	Regulated	Q-value	Description	References
MAC_07957	4.006674414	Up	0.00016526	Lactate dehydrogenase	[55-57]
MAC_05898	3.542727315	Up	3.77E-12	Aminoadipate-semialdehyde dehydrogenase, putative	[58]
MAC_05899	2.398991837	Up	0.000638775	ATP-binding cassette sub-family B member 5	[59]
MAC_09405	2.271015018	Up	4.75E-109	Sorbitol dehydrogenase	[60]
MAC_07958	1.986496532	Up	2.02E-08	Tyrosinase, putative	[61]
MAC_02819	1.913732853	Up	2.76E-212	2,3-dihydroxybenzoic acid decarboxylase dhbd	[62]
MAC_00175	1.474845169	Up	4.40E-150	Glucose dehydrogenase, putative	[63]
MAC_06622	1.428907415	Up	1.74E-05	Aspartic protease	[64]
MAC_04042	1.405343517	Up	1.78E-139	Oxidoreductase, 2-nitropropane dioxygenase family, putative	[65]
MAC_04217	1.372152788	Up	0	Heat shock protein 30	[66]
MAC_01184	1.352512998	Up	4.08E-57	Ribose 5-phosphate isomerase	[67]
MAC_09768	1.086378419	Up	1.56E-60	Amino-acid permease inda1	[68]
MAC_07959	1.072525094	Up	0	Hydantoinase/oxoprolinase, putative	[69]
MAC_02934	-1.031017876	Down	1.79E-14	Glucose-methanol-choline oxidoreductase	[70]
MAC_02571	-1.097808903	Down	0	Endoglucanase, putative	[71]
MAC_06606	-1.334362504	Down	6.23E-65	GMC oxidoreductase, putative	[73]
MAC_06944	-1.345310914	Down	7.84E-07	P450 monooxygenase	[74]
MAC_08413	-1.387604525	Down	8.43E-07	Alpha/beta hydrolase fold family protein	[75]
MAC_08685	-2.062134744	Down	2.66E-40	Flavin containing amine oxidase, putative	[76]
MAC_05384	-2.067526528	Down	6.04E-42	Laccase	[77]
MAC_05385	-2.524707046	Down	2.27E-77	Conidial pigment polyketide synthase pksp/Alb1	[78]
MAC_09145	-4.947521896	Down	3.55E-08	Carboxylesterase family protein	[79]
